# Decoding Death in Complexity: A Forensic Case Series on Trauma, Healthcare-Associated Infections, and Chronic Diseases

**DOI:** 10.7759/cureus.96734

**Published:** 2025-11-13

**Authors:** Georgiana-Denisa Gavriliță, Ștefania Ungureanu, Alina-Cristina Pașca, Ecaterina Dăescu, Alexandra Enache

**Affiliations:** 1 Doctoral School, "Victor Babeș" University of Medicine and Pharmacy Timișoara, Timișoara, ROU; 2 Forensic Medicine, Institute of Forensic Medicine Timișoara, Timișoara, ROU; 3 Ethics and Human Identification Research Center, Department of Neuroscience, Discipline of Forensic Medicine, Bioethics, Deontology and Medical Law, "Victor Babeș" University of Medicine and Pharmacy Timișoara, Timișoara, ROU; 4 Discipline of Forensic Medicine, Bioethics, Deontology and Medical Law, Department of Neuroscience, "Victor Babeș" University of Medicine and Pharmacy Timișoara, Timișoara, ROU; 5 Forensic Medicine, "Pius Brânzeu" County Emergency Clinical Hospital Timișoara, Timișoara, ROU; 6 Discipline of Anatomy and Embryology, Department I, "Victor Babeș" University of Medicine and Pharmacy Timișoara, Timișoara, ROU; 7 Pathology, Institute of Forensic Medicine Timișoara, Timișoara, ROU; 8 Ethics and Human Identification Research Center, Department of Neuroscience, Discipline of Forensic Medicine, Bioethics, Deontology and Medical Law, “Victor Babeș” University of Medicine and Pharmacy Timișoara, Timișoara, ROU; 9 Discipline of Forensic Medicine, Bioethics, Deontology and Medical Law, Department of Neuroscience, “Victor Babeș” University of Medicine and Pharmacy Timișoara, Timișoara, ROU

**Keywords:** causality, causal relationship, chronic diseases, healthcare-associated infections, process of death, trauma

## Abstract

Due to the nature of the profession, healthcare-associated infections (HAIs) are a prevalent problem in forensic practice, especially in patients who have been hospitalized following severe injuries that required extended hospital stays, which often include surgery. Nevertheless, some of these patients also have underlying medical conditions. When a patient passes away and a complex pathology is discovered, it can be challenging to determine the cause of death and how each condition contributed to the death process. We present three cases from our practice that illustrate our approach to managing cases with multifactorial pathology.

## Introduction

In forensic practice, a large part of the activity is dedicated to traumatic injuries, both in living and deceased patients, and the issue of healthcare-associated infections (HAIs) is common because of the nature of the work, particularly in patients who are hospitalized after suffering serious traumatic injuries that require lengthy hospital stays, which often include surgery. Additionally, some patients also have pre-existing chronic diseases, which can influence their medical evolution. Therefore, many factors need to be carefully analyzed by the forensic doctor when establishing the cause of death [1].

When working with deceased patients, causality is essential in forensic medicine because the patient's death is always the first effect we face. Determining the conditions and factors that contributed to the cause of death is another benefit of causality. This aids in developing a rational, reasonable, and impartial answer to queries raised by the judicial system. A forensic expert report, which covers general causation (whether an exposure can cause the injury observed in an individual), particular causation (if the exposure did cause the injury observed in an individual), or both, is typically required to be presented to the court as the outcome of a causal analysis [2]. Considering contemporary scientific advancements, forensic opinions must be supported by compelling evidence and delivered after a specialist’s analysis, in which professional experience plays a key role.

A concept known as the "causal relationship" describes the relationship between the negative consequences of an action or inaction and the outcomes that result. We refer to a primary or direct causal relationship when the trauma and its effects cannot be changed or when the trauma's effects are lessened by preceding or coexisting circumstances. When a complication that is thought of as a secondary cause that otherwise would not have occurred in evolution develops between the shock and the effect, it is referred to as a secondary or indirect causal relationship. This might result in a far more severe post-traumatic consequence or even the victim's death. A tertiary or associative causal relationship is observed when the traumatic factor, a pre-existing pathological condition, and a post-traumatic consequence are all present simultaneously with the effect [1].

In cases of multifactorial disease, the causal relationship between trauma and death needs to be carefully explored, taking into account all of the patient's conditions. A few medico-legal cases, including non-traumatic illnesses, HAIs, and traumatic injuries, were examined by us to demonstrate how a forensic physician interprets all the conditions and causes that led to death and elaborates conclusions regarding the type of death, the cause of death, the causal relationship between traumatic injuries and death, and the role of the patient's pre-existing comorbidities in the process of death. We could evaluate the mortality risk associated with each patient's condition (traumatic injury, pre-existing ailment, HAI) and ascertain the cause of death through a classification of these conditions. We established three categories for traumatic injuries: high-risk injuries, which occur when there is a substantial impairment in the functioning of the respiratory, circulatory, or central nervous systems, either separately or in combination; moderate-risk injuries, which are dangerous but may improve with the right care and supervision; and low-risk injuries. We classified patients' chronic illnesses as either decompensated pathogenic conditions, which are uncontrolled by medication or other care and have an immediate elevated risk of death, or compensated pathogenic conditions, which lack an immediate risk of death. Three categories were created to classify HAIs: limited nosocomial infections, which have a low risk of becoming fatal; spread (bacteremia); and widespread (septicemia, sepsis, severe sepsis, and septic shock) with a higher risk of death (bronchopneumonia). The influence of HAIs, if traumatic injuries had an adverse evolution due to the patient's pre-existing pathologies, whether traumatic injuries aggravated or decompensated pre-existing chronic diseases, and the interaction between the patient's illnesses, were all carefully investigated. Subsequently, by documenting the time of death, we may evaluate the probability that each illness will culminate in mortality. In the end, we could identify the cause of death and the relationship between the patients' traumatic injuries and their demise.

## Case presentation

Three autopsy cases with traumatic injuries, pre-existing pathological conditions, and nosocomial infections were selected from the casuistry of the Timișoara Institute of Forensic Medicine between 2015 and 2024. The examples were selected randomly based on the pathology and cause of death to illustrate three distinct scenarios about the causal relationship between trauma and death. The cases listed below satisfied three requirements for inclusion: they had a chronic condition that existed at the time of admission; they experienced a traumatic injury that required hospitalization; and they developed an HAI while in the hospital that was either clinically or postmortem identified. We reviewed the patients' medical records and autopsy reports, and we tracked the patients' clinical development, autopsy results, and cause of death. To accurately demonstrate and substantiate a causal relationship between the victim's death and the traumatic injuries, the following factors were taken into account: each patient's age and gender; the traumatic injuries according to the autopsy findings (location, macroscopic appearance, healing stage, and results of the histopathological examination); the length of hospital stays; pre-existing chronic diseases (type, severity, age, and both clinical and autopsy diagnosis); and the diagnosis of nosocomial infection (clinical diagnosis, microbiological tests, severity, treatment, response to treatment, autopsy findings, and clinical diagnosis).

Case 1

A 74-year-old male was admitted to the hospital after suffering spinal trauma after a fall from a height. A compression fracture of the third lumbar vertebral body (L3) was discovered during imaging investigations at the time of admission. This fracture resulted in substantial retropulsion within the posterior wall canal and a loss of 60-70% of the original height, causing severe canal stenosis at this level. The patient was admitted to the Neurosurgery Department of Timișoara's Emergency County Hospital Pius Brânzeu (ECHPBT), despite the fact that no surgery was performed. Chronic lymphoid leukemia, chronic respiratory failure, chronic obstructive pulmonary disease, pulmonary emphysema, heart failure (New York Heart Association (NYHA) II), degenerative mitral regurgitation, mild functional tricuspid regurgitation, type II diabetes, hypothyroidism, mixed dyslipidemia, condition following infection with SARS-CoV-2, and bipolar affective disorder were among the patient's significant pathological conditions. Nine days following admission, the patient's respiratory condition suddenly deteriorated, and oxygen saturation dropped to 74%. A fine pleural fluid layer and bilateral basal alveolar condensation areas were visible on the thoracic CT. Following consultation with an infectious disease specialist, nosocomial bronchopneumonia was diagnosed, further testing was ordered, and antibiotic treatment was started. The patient passed away the following day, and he was brought to Timișoara's Institute of Forensic Medicine for a medico-legal autopsy.

**Figure 1:**
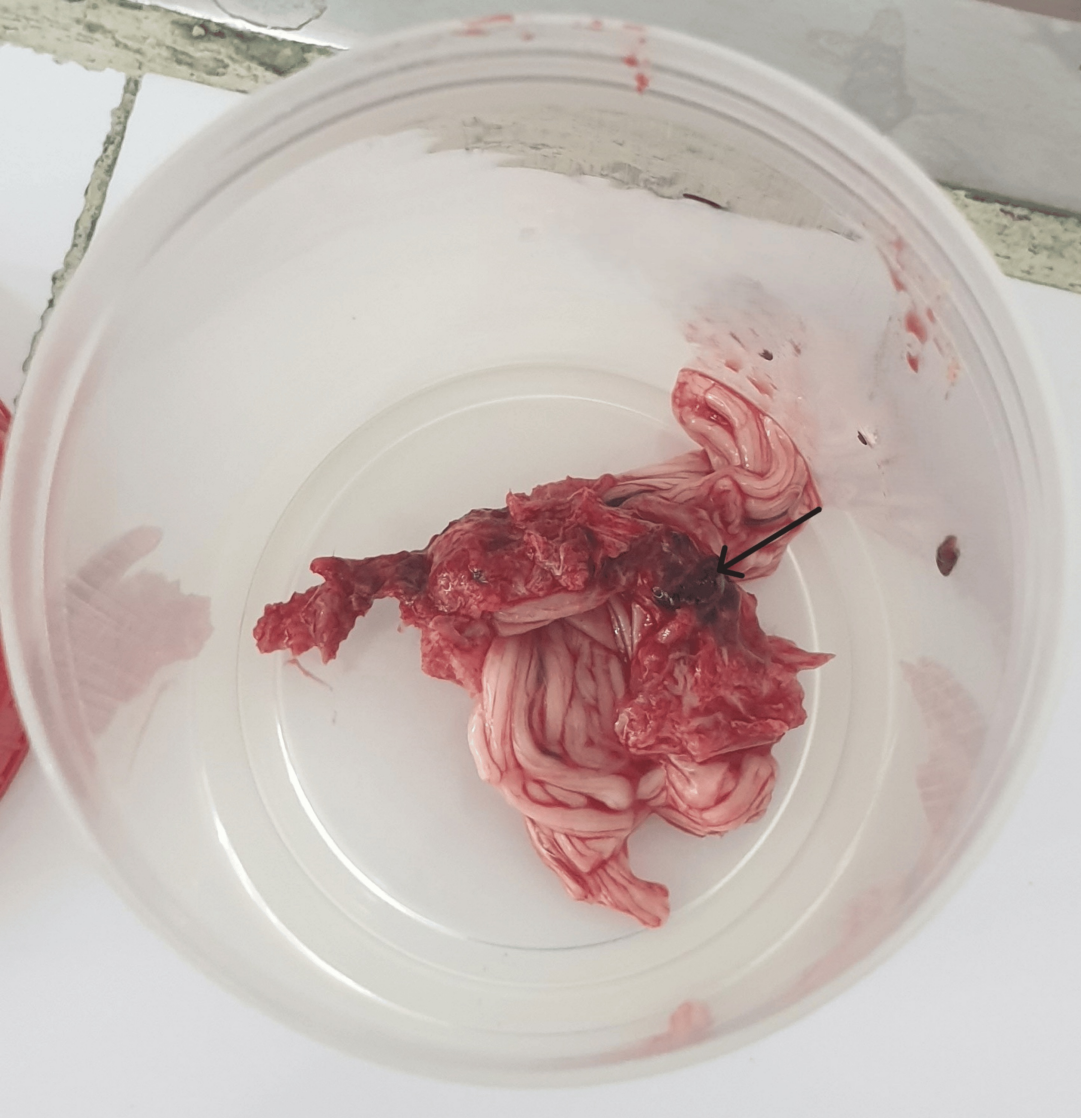
Cauda equina fragment extracted during the autopsy Macroscopic view of a cauda equina fragment at the level of the L3 vertebra showing hemorrhagic region (arrow)

**Figure 2:**
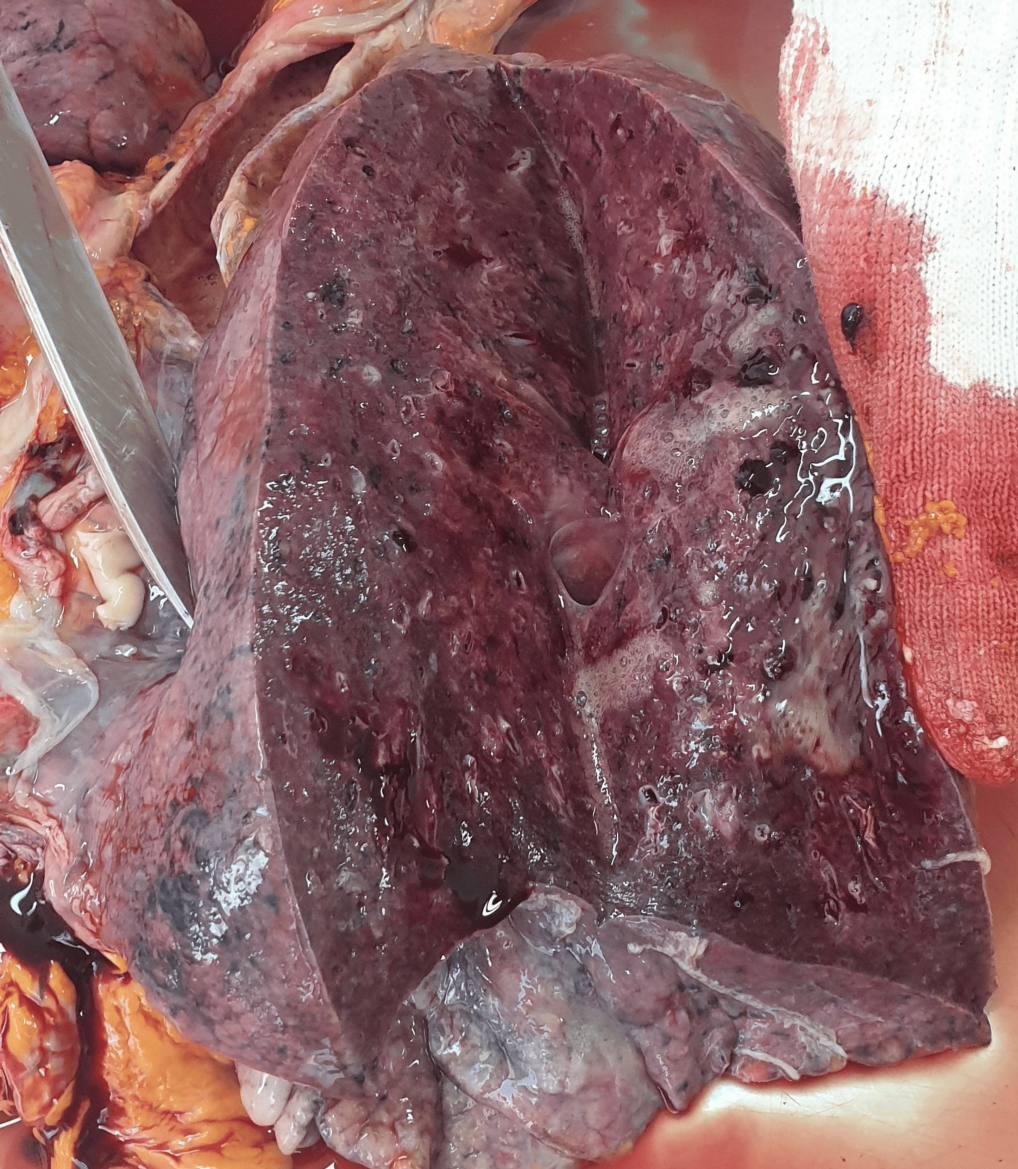
Sectioned lung at autopsy Macroscopic aspect of bronchopneumonia: consolidation of the lung with edema

The autopsy revealed a comminuted fracture of the L3 vertebral body and cauda equina injury (Figure 1). Additionally, rib and sternum fractures were found as a result of cardiopulmonary resuscitation procedures. Bronchopneumonia (Figure 2), mild serous pleurisy, myocardial fibrosis, a right renal cyst, and systemic atheroma were all visible upon macroscopic examination of the internal organs.

**Figure 3:**
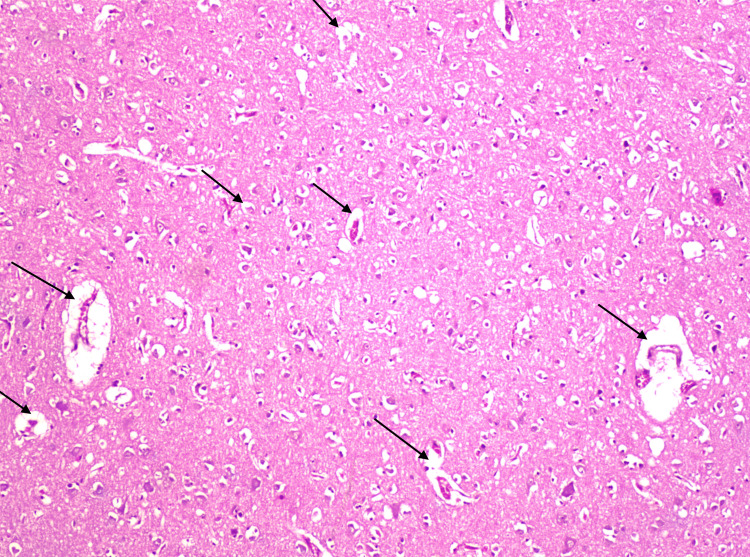
Histological section of the brain (HE, x 100) Optically transparent regions (arrows) surrounding the blood vessels and cells, indicating cerebral edema progression

**Figure 4:**
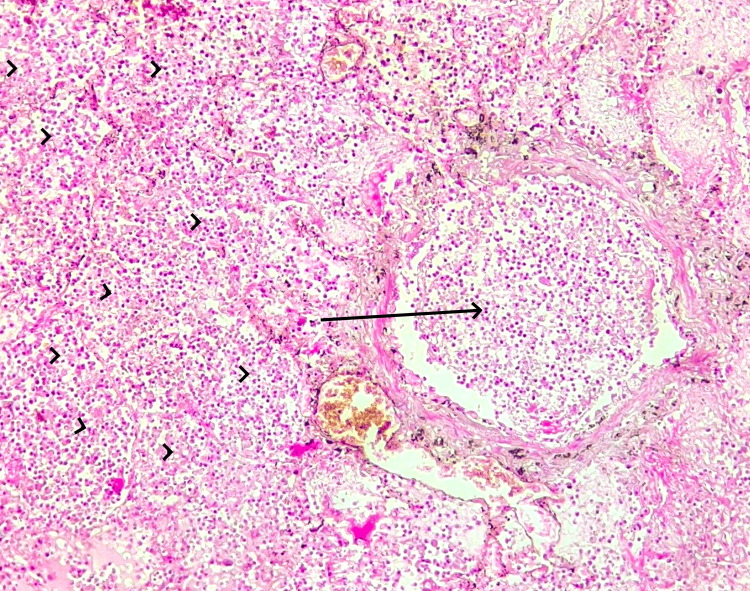
Histological section of the lung (HE, x100) Microscopic findings on lung tissue: bronchiola with acute bronchiolitis (arrow); leukocytic alveolitis (arrowheads)

**Figure 5:**
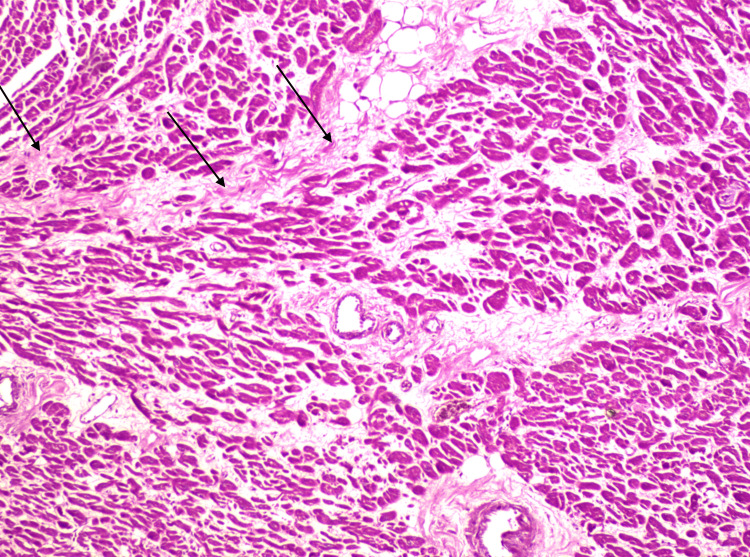
Histological section of the heart (HE, x 100) Myocardial section showing aspects of interstitial infiltrative fibrosis (arrows)

**Figure 6:**
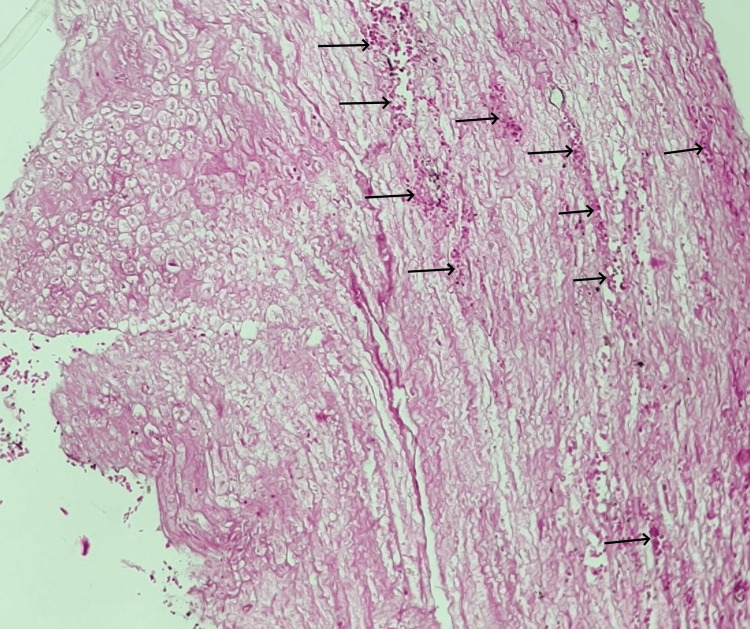
Histological examination of the cauda equina fragment Microscopic findings on cauda equina tissue showing blood infiltrates (arrows)

In addition to the medico-legal autopsy, a histological analysis of the tissues taken from the brain, lung, heart, and cauda equina revealed cerebral edema (Figure 3), bronchopneumonia (Figure 4), cardiac fibrosis (Figure 5), and blood infiltrates, edema, and fibrin deposits at the level of the spinal nerves (Figure 6). Paraffin was used to implant tissues, and 3 μm slides were cut and stained with hematoxylin and eosin.

We highlight the following points in relation to this case: a fracture of the L3 vertebral body, which is a non-life-threatening injury with a minimal chance of mortality, was the reason for the patient's hospital admission. In addition, he had several non-traumatic conditions, including pulmonary pathology, chronic lymphoid leukemia, type II diabetes, hypothyroidism, and cardiovascular disorders. Despite being compensated under treatment, these disorders could compromise the patient's immunity and provide an environment that is conducive to infections, particularly in the pulmonary pathology. According to the medical records and autopsy findings, the patient developed healthcare-associated bronchopneumonia while in the hospital. The autopsy and histopathological findings established that bronchopneumonia was the proximate cause of death, evolving as a hospital-acquired infection secondary to the initial spinal injury. Bronchopneumonia may also have been exacerbated by the patient's pre-existing pathological conditions. The causal relationship between trauma and death is indirect, as the trauma necessitated hospitalization, where the fatal infection was acquired. 

Case 2

Six weeks after suffering a fracture to her right femur, a 61-year-old woman was taken to the ECHPBT's orthopedic department. At the time of the trauma, the patient refused surgery, choosing to get conservative treatment at home instead. The patient had a right thigh that was edematous, a lower right limb that was abducted two millimeters, an early-stage pressure ulcer on the right buttock, and pain in the right hip and total impotence when she was admitted to the hospital. On femur radiography, an unhealed spiral comminuted fracture was seen in the right proximal region of the femur. The patient's personal pathological history included chronic ischemic heart disease, depression, and grade 3 obesity. Two days after admission, open reduction of the femur fracture and internal fixation with a gamma implant, irrigation, drains, and sutures in anatomical planes were performed. On the same day after surgery, the patient developed tachypnea, dyspnea, and hemodynamic instability. Because of the patient's sudden respiratory decrease, pulmonary thromboembolism was suspected; however, imaging investigations did not confirm this diagnosis. The patient's health worsened, and she died two days after being admitted to the ICU, where she received the proper care and close observation. The next day, she was brought to Timișoara's Institute of Forensic Medicine for a medico-legal autopsy.

**Figure 7:**
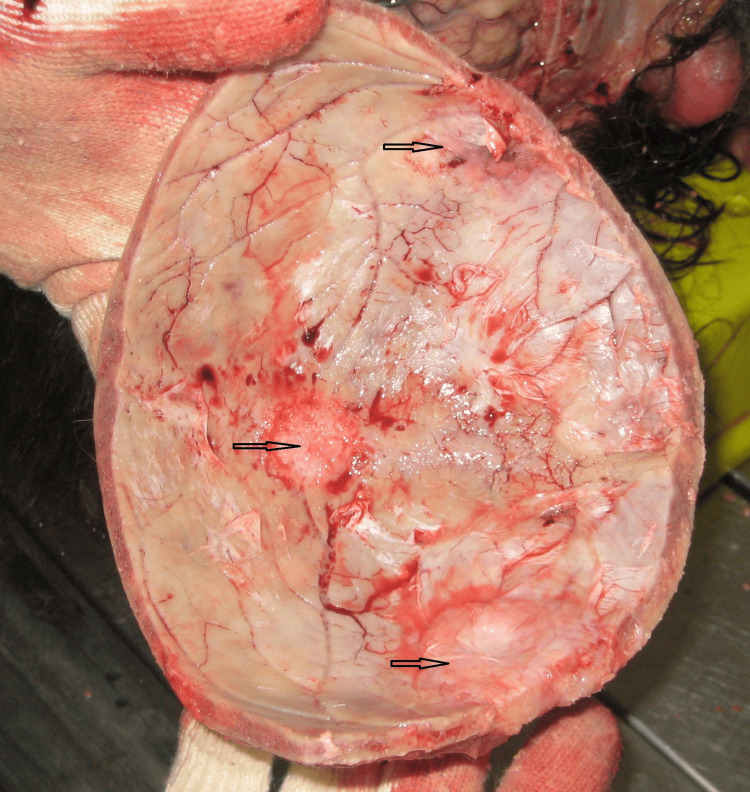
Skull fragment cut during autopsy Macroscopic view of some cranial bone abnormalities resembling metastases (arrows)

The autopsy revealed the following macroscopic findings: cirrhosis, cardiac fibrosis, incipient pneumopathy, obesity, surgical incision, and cranial bone anomalies that resembled metastases (Figure 7).

**Figure 8:**
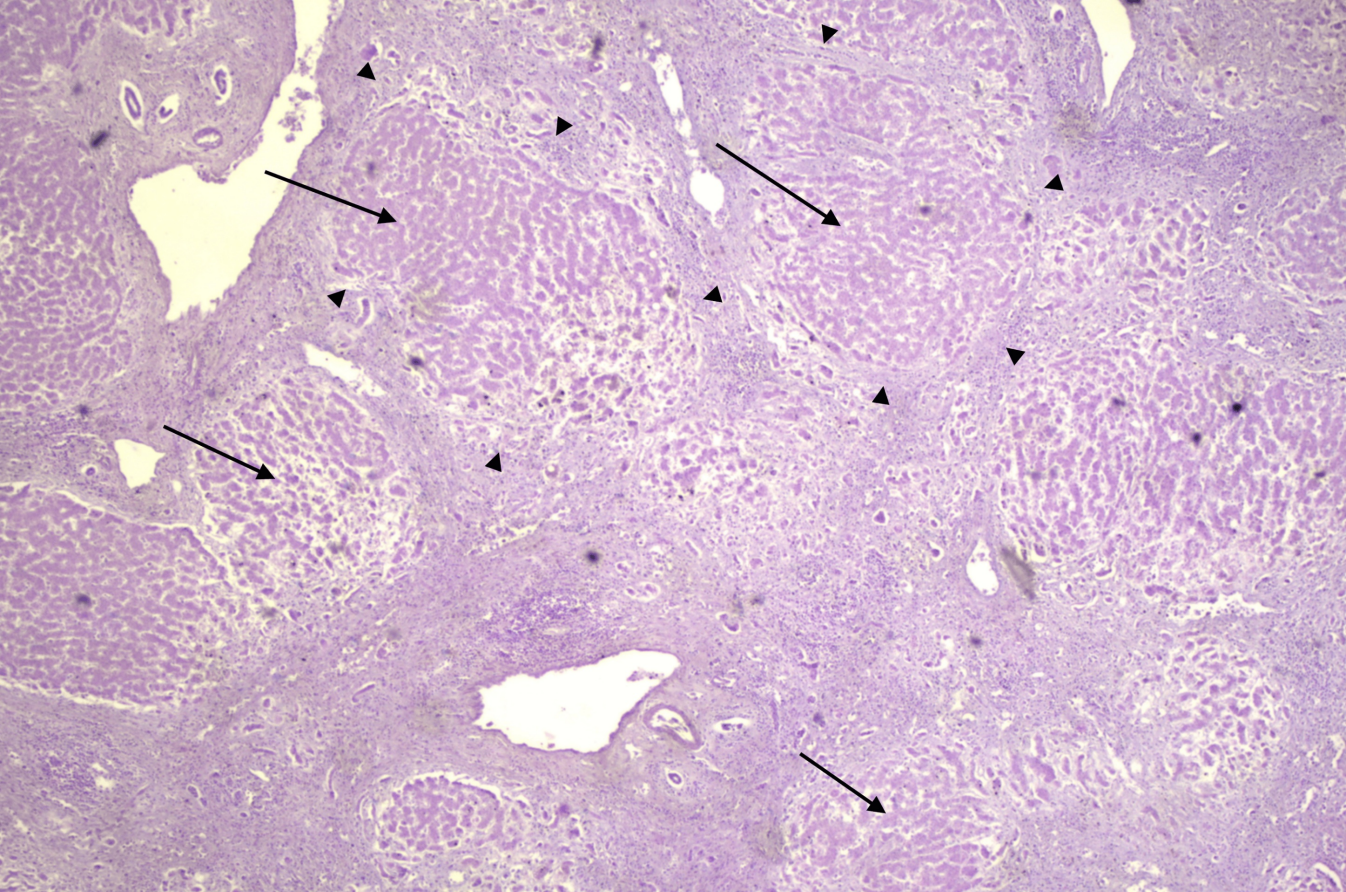
Histological section of the liver (HE, x40) Wide septa of fibrosis, associated with lymphocytic inflammatory infiltrate and bile duct proliferation (arrowheads), delineating numerous regenerative nodules (arrows)

**Figure 9:**
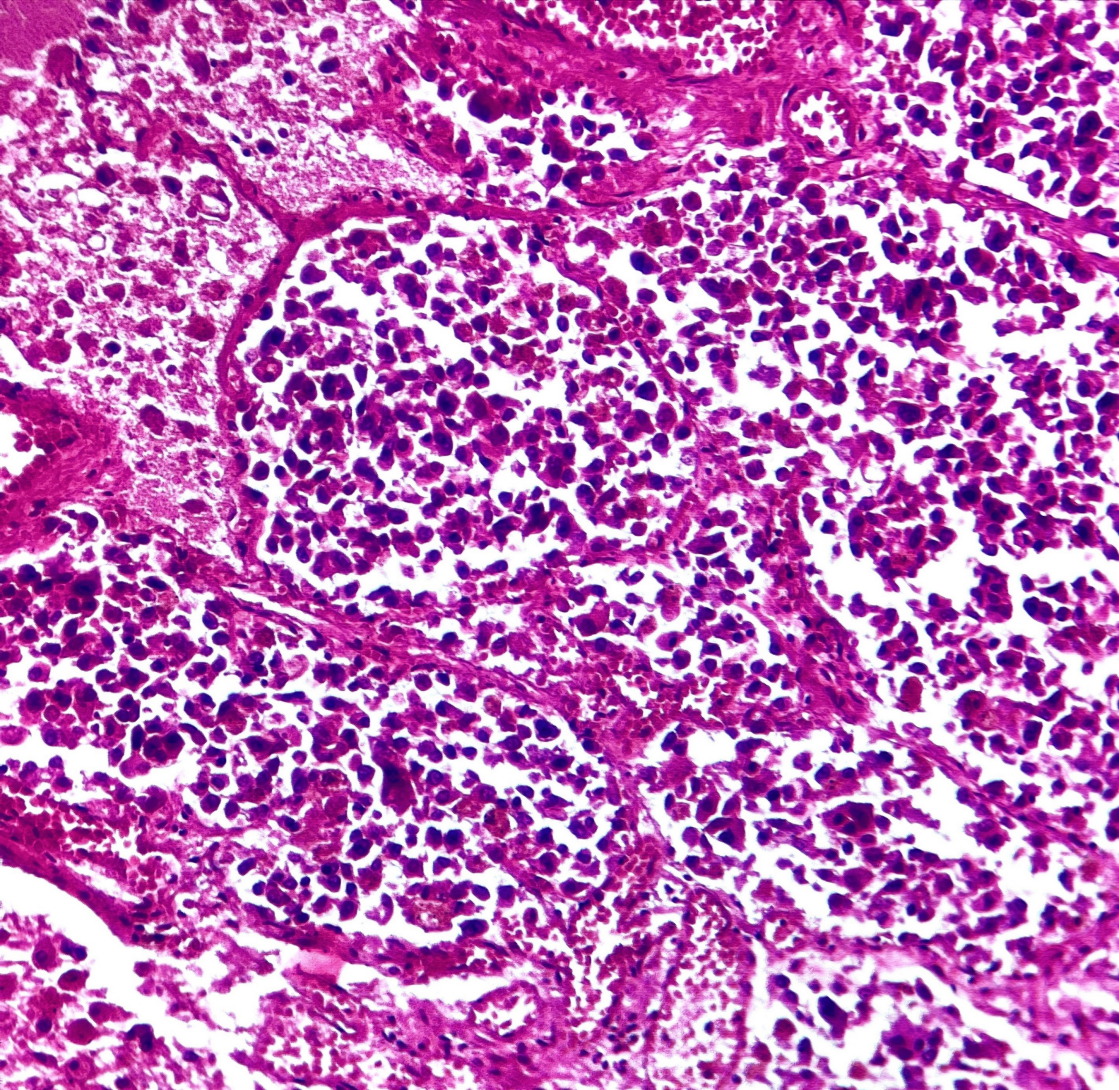
Histological section of the lung (HE, x100) Microscopic findings on lung tissue: pulmonary non-small cell carcinoma spread through the air spaces

Histological analysis of the liver and lung samples taken during the autopsy revealed micronodular cirrhosis (Figure 8) and pulmonary non-small cell carcinoma (Figure 9). Tissues were implanted in paraffin, and 3 μm slides were cut and stained with eosin and hematoxylin.

After evaluation, we emphasize the following aspects regarding Case 2: even while a femur fracture is not fatal, prolonged bed rest might raise the risk of lung infection, especially in older patients. The patient also had risk factors, such as depression and obesity. However, the lung piece's histological analysis showed aggressive non-small cell carcinoma, which explains the patient's declining respiratory condition. Non-small-cell lung cancer (NSCLC) is one of the most frequent cancer types and is responsible for most cancer-related deaths worldwide. NSCLC accounts for about 80 to 85 out of 100 lung cancer cases.

Adenocarcinoma, squamous cell carcinoma, and big cell carcinoma are the three primary varieties. Investigations using tissue-sparing immunohistochemistry are essential for determining the cell type of a non-small cell carcinoma [3]. This is a unique case because the patient did not exhibit any typical symptoms of pulmonary oncological disorders, such as hemoptysis, weight loss, or breathing difficulties. Additionally, the patient had no known lung issues at the time of admission, and no chest imaging tests were conducted. The femur fracture was not a direct cause of death, and we determined that the death was non-violent and caused by lung cancer.

Case 3

**Figure 10:**
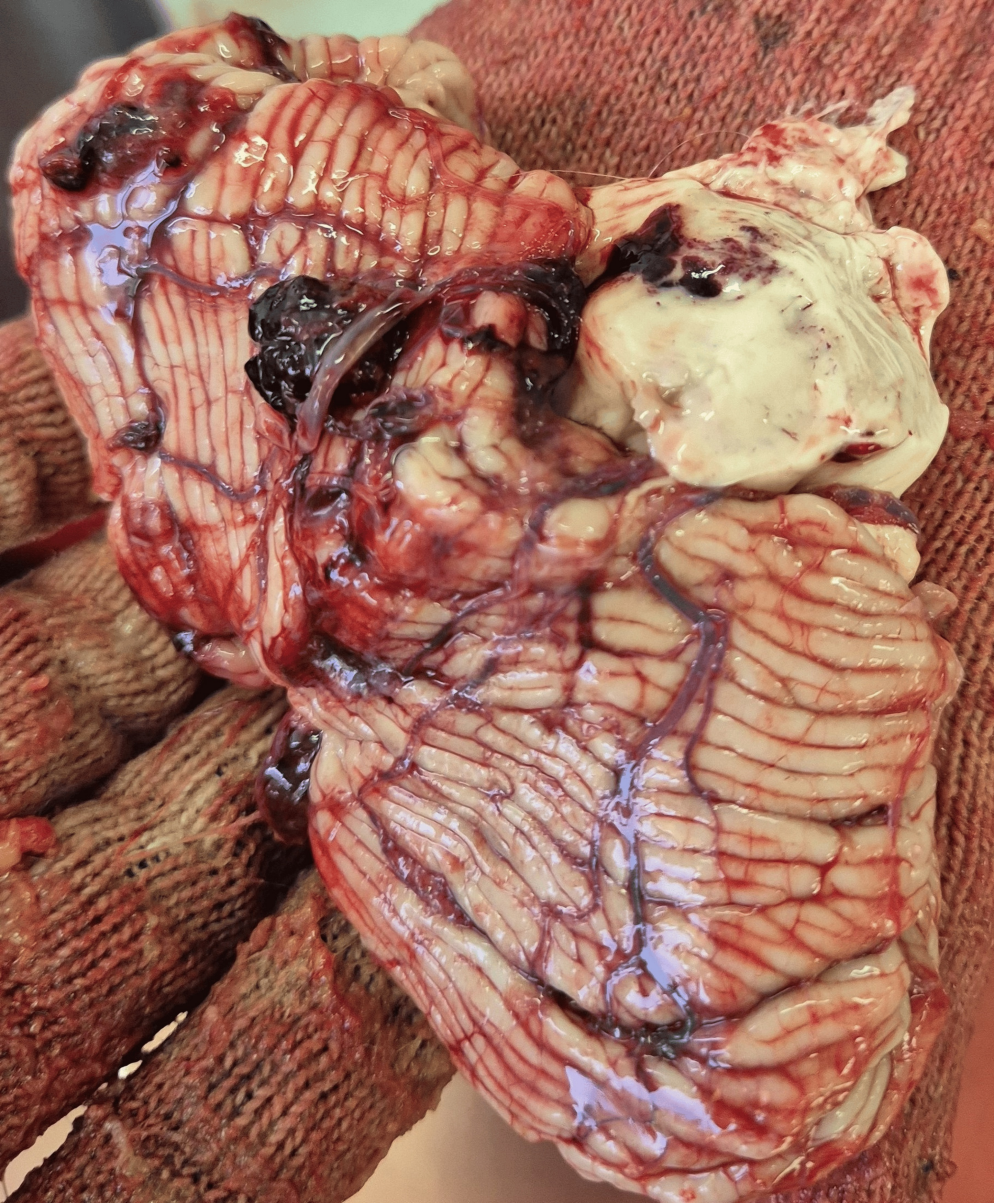
Sectioned brainstem at autopsy Hemorrhagic foci and contusion area

A 68-year-old male was taken to the ECHPBT Emergency Unit with a severe head injury of unknown origin. Upon arrival, imaging studies revealed an acute subdural hematoma in the left cerebral hemisphere and a right temporal and parietal fracture. The left frontal-temporal-parietal bone flap and drainage were used to remove the hematoma. After that, the patient was admitted to the ICU at ECHPBT. His Glasgow Coma Score ranged from 6 to 10, and he was going through periods of progress and regression. He developed sepsis, nosocomial bronchopneumonia, and several bedsores. *Proteus mirabilis* and *Staphylococcus aureus* were found in the microbiological testing. After 41 days of hospitalization, the patient died. There were no pre-existing pathological conditions listed in the hospital's medical records of the patient. An autopsy performed at Timișoara's Institute of Forensic Medicine revealed a surgical incision, venous puncture marks, decubitus ulcers, moderate cerebral, coronary, and aortic atherosclerosis, pericranial hemorrhagic infiltrates, a craniectomy hole, a skull fracture, cerebral edema, cerebral and brainstem hemorrhagic contusions (Figure 10), pulmonary edema, bronchopneumonia, moderate pleurisy, left ventricular hypertrophy, myocardial fibrosis, and kidney failure.

**Figure 11:**
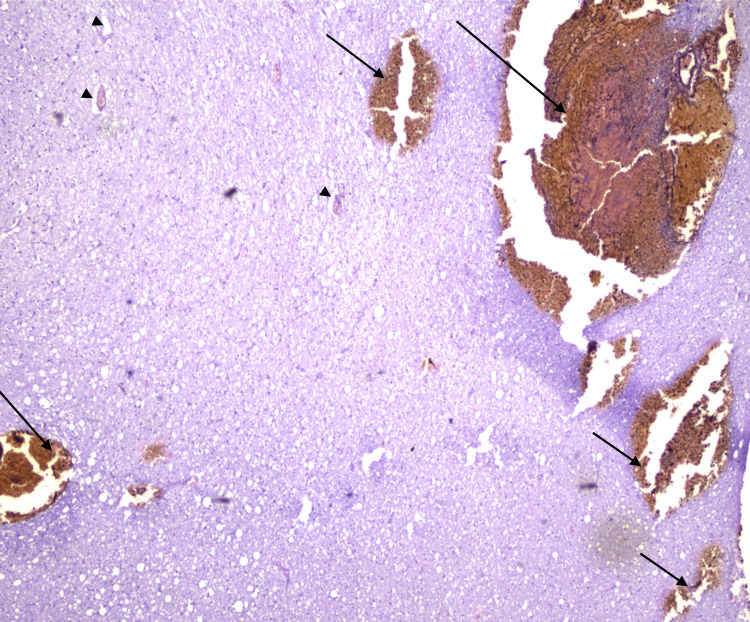
Histological section of the brain (HE, x40) Optically clear spaces (arrowheads) formed around the blood vessels and cells, indicative of the development of cerebral edema and intraparenchymal hemorrhagic foci (arrows)

**Figure 12:**
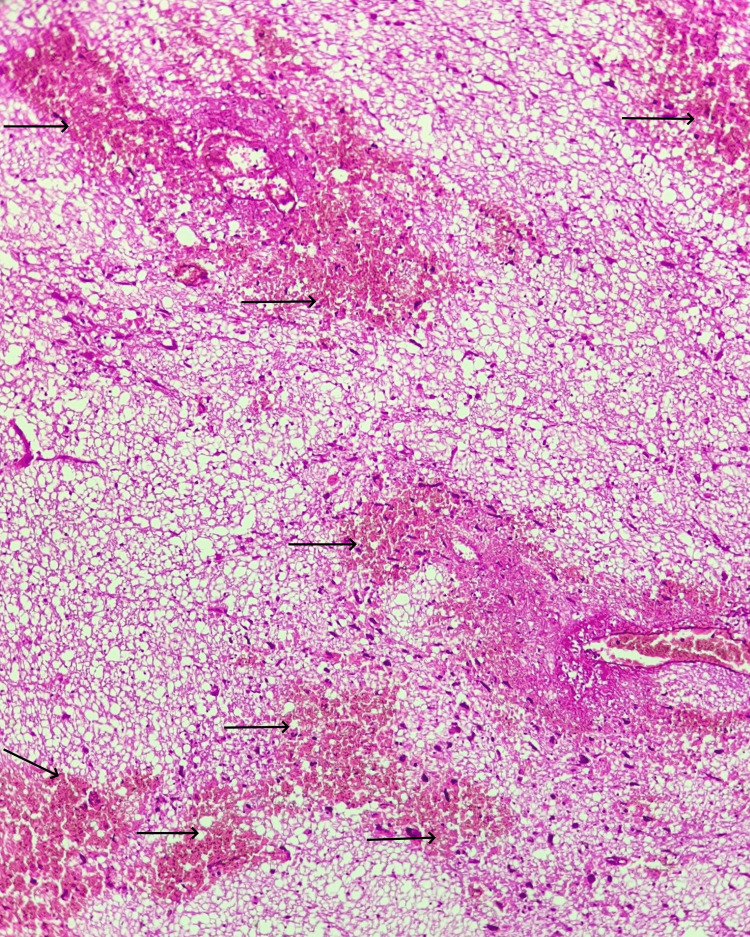
Histological section of the brainstem (HE, x40) Microscopic view of the brainstem tissue with hemorrhagic areas (arrows)

**Figure 13:**
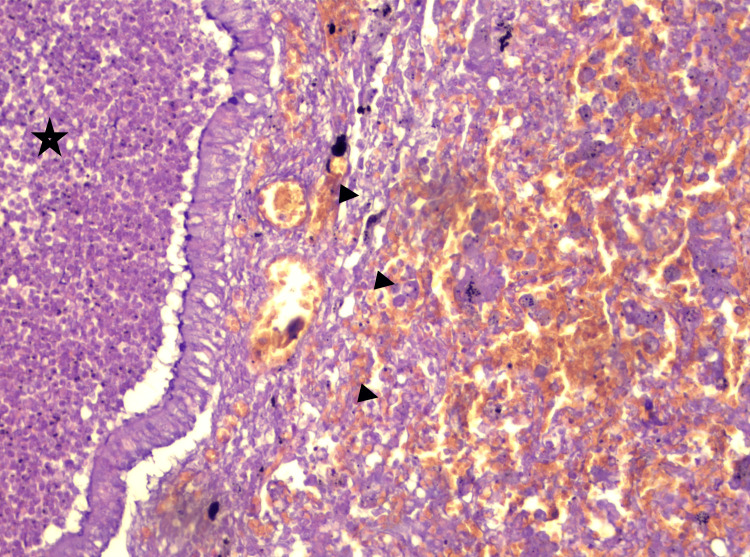
Histological section of the lung (HE, x100) Purulent exudate in the lumen of a bronchiole (star). Hematoleukocytic alveolitis (arrowheads)

**Figure 14:**
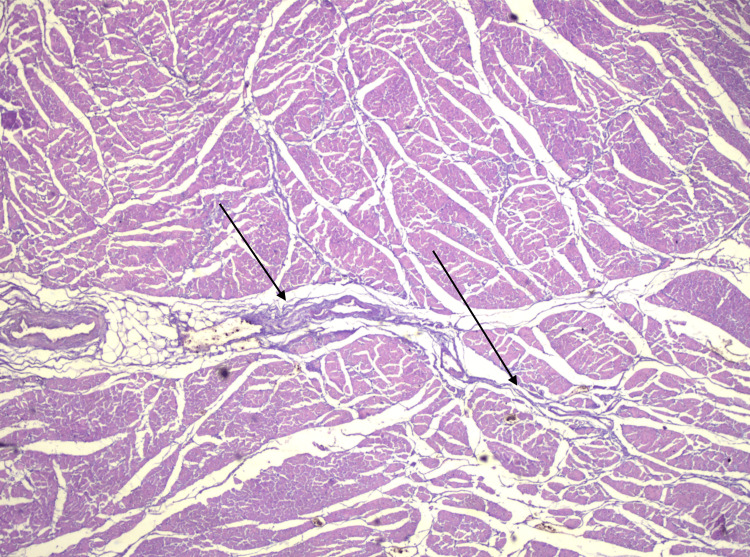
Histological section of the heart (HE, x40) Discrete aspects of myocardial interstițial fibrosis, predominantly perivascular (arrows)

**Figure 15:**
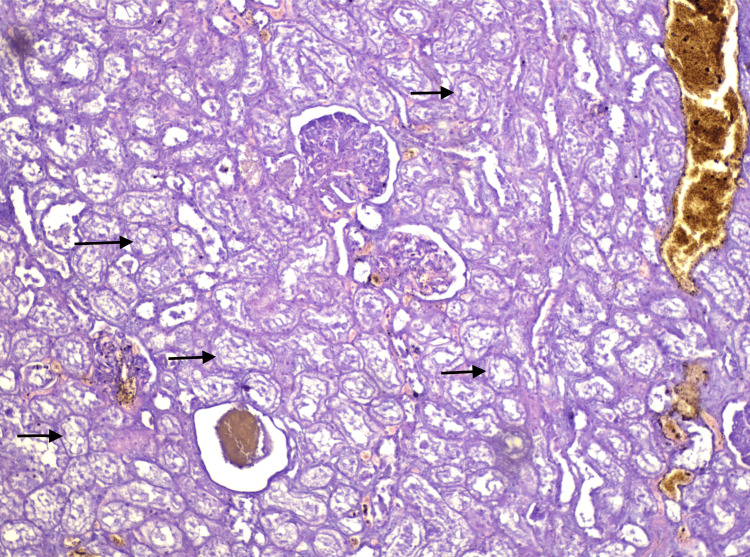
Histological section of the kidney (HE, x100) Acute tubular necrosis, subject to autolytic changes (arrows)

Histopathological examination of the lung, heart, kidney, brain, and brainstem tissues obtained during the medico-legal autopsy showed systemic arteriofibrosis, hemorrhagic foci and perivascular hematous extravasations in the cerebral white matter, cerebral edema (Figure 11), hemorrhagic foci in the brainstem (Figure 12), acute bronchopneumonia (Figure 13), mild cardiac fibrosis (Figure 14), and acute tubular necrosis in the epithelia of the renal convoluted tubules (Figure 15). After implanting tissues in paraffin, 3 μm slides were cut and stained with hematoxylin and eosin.

These results indicate that the patient's death was violent and caused by a head injury that involved a fractured skull, an acute subdural hematoma that was surgically evacuated, and cerebral and brainstem contusions. Given the severity of traumatic brain injury, we recognized that the patient's pre-existing cardiac conditions (myocardial fibrosis, left ventricular hypertrophy) and the HAIs (sepsis, bronchopneumonia) did not contribute to death.

The cases presented above illustrate three different situations of patients with traumatic injuries, pre-existing chronic conditions, and HAIs in order to exemplify different types of causal links between trauma and death. To identify the cause of death in patients with multifactorial pathology, each disease must be thoroughly assessed independently, and the condition with the highest risk of death must be selected. Consideration of all medical problems is necessary due to the substantial legal ramifications of establishing a causal link. Case 1 emphasizes how important it is to provide nosocomial infections in trauma patients with extra attention, particularly when the injuries do not carry a significant risk of death, and illustrates an indirect causation between trauma and death. The patient in Case 2 had a lung tumor that had not been diagnosed, which shows how important it is to do a full clinical and paraclinical assessment of the patient in addition to the serious injuries they bring to the hospital. It also shows a death that is not related to trauma. Case 3 presents a scenario in which the patient's traumatic injuries are so severe that they would be the cause of death in any case, even though they came on top of pre-existing chronic illnesses and nosocomial infections, and emphasizes a direct causal relationship between trauma and mortality.

## Discussion

The most frequent main objective of forensic medical analysis is to give legal factfinders proof of the causal connection between an alleged action by the accused (in a criminal case) or defendant (in a civil case) and a medically observed harmful outcome (injury, disease, or death), even though the practice, definitions, and applications of forensic medicine differ significantly by geographic and jurisdictional region [2]. 

Trauma remains a significant public health issue worldwide, despite decades of substantial progress in prevention and treatment. Injuries are estimated to result in five million fatalities per year [4]. The interpretation of autopsies in trauma fatalities is crucial for trauma research and specifically for ongoing quality enhancement initiatives. The precision and complexity of a forensic pathologist's assessment of injury causation may vary from that of a medical interpretation of the injuries. The principal difficulty in investigating causation is its inability to be directly observed or measured. Forensic medical practitioners must assess whether the antecedent contributed to an outcome (death or injury) when confronted with one or more suspected causal agents [5]. According to a set of triage criteria for treatment, trauma cases are classified as minor or significant in routine clinical practice. Since a severe trauma may have little or no forensic value, while a minor trauma that can be readily disregarded may have a significant medico-legal impact, such a classification will not be applicable in trauma forensics. Thus, in trauma forensics, medical professionals must categorize trauma in a way that is suitable and intelligible to the legal system and that may indicate the cause [6].

Globally, traumatic brain injury (TBI) is the primary cause of death and disability in individuals under 45 years of age, and it is also a significant cause of mortality in other age groups [7]. Fractures of the limbs and pelvis, as well as damage to the thorax, abdomen, and spinal cord, ensue [8]. From a neuropathological standpoint, TBI is categorized into two principal stages: primary damage, occurring at the moment of injury and encompassing scalp abrasions, skull fractures, surface contusions, brain abrasions, diffuse axonal injuries, and intracranial hemorrhage; and secondary damage, resulting from intricate processes initiated at the time of injury but not immediately evident, including brain damage due to elevated intracranial pressure, ischemia, edema, and infection. The objective of the autopsy is to identify both primary and subsequent cerebral damage [7]. One of the concerns raised by intracranial injuries is that the majority of patients may be at an age where they are more susceptible to chronic illnesses. Secondly, the management of patients with intracranial injuries is complicated by the common occurrence of concomitant injuries in other regions of the body. Even if they endure surgery, these patients may succumb to bodily decompensation and problems arising from their injuries. Certain individuals perish due to complications from their injuries, such as pneumonia, rather than the injuries themselves [9]. Spinal cord injury (SCI) results not only in motor and sensory deficits but also in autonomic dysfunctions because of the disruption between higher brain centers and the spinal cord. Autonomic dysfunction can affect cardiovascular, respiratory, urinary, gastrointestinal, thermoregulatory, and sexual functions [10]. An important and potentially life-threatening secondary complication of SCI is pressure ulcers. They can lead to further functional disability and fatal infections, while surgical interventions can be required [11]. The evaluation of any anterior pathological condition of the spine is essential in situations of SCI. Medico-legal concerns become more complex when pre-existing conditions are present [12]. The differential diagnosis of a pathological bone fracture due to illness rather than trauma necessitates careful evaluation. In polytraumatized patients, lower limb fractures, particularly femur fractures, are predominantly attributed to high-energy trauma; yet, low-energy trauma in elderly individuals or compromised bones can also result in these injuries. In the geriatric population, important risk factors for in-hospital mortality include a longer period from injury to operation, lower preoperative hemoglobin level and estimated glomerular filtration rate (eGFR), and greater preoperative white blood cell count [13].

The patient's chronic conditions may fundamentally disrupt the trajectory of a traumatic injury by generating a subtle field that amplifies the morpho-functional resonance of the lesion, thereby hindering effective treatment and prolonging recovery. Preexisting chronic medical disorders appear to elevate the mortality risk for elderly trauma patients [14]. Furthermore, several conditions, such as diabetes, morbid obesity, and chronic venous insufficiency, increase the susceptibility of individuals to pathogenic microorganisms, hence increasing the risk of nosocomial infections. Elderly people exhibit twice the death rate and increased morbidity compared to younger ones for comparable damage levels, due to substantial comorbidities and associated treatments. Individuals aged over 65 are increasingly impacted and are at a higher risk of mortality due to medical issues occurring late in their hospital stay [15].

Despite receiving adequate therapy during hospitalization, the effects of the traumatic agent may be worsened by potential infection complications. Moreover, the patient's advancement might sometimes be negatively influenced by preexisting pathological conditions. In ascertaining the cause of death, these aspects must be evaluated, as they significantly influence the death process. The impact of infection issues after trauma on in-hospital mortality often varies according to the extent of the injury, and it still requires great monitoring even in patients with moderate dysfunctions [16]. Even with a more thorough understanding of the pathophysiological alterations brought on by trauma and surgery, pulmonary problems continue to be a leading cause of morbidity and death. The patients' pre-existing lung diseases render them more susceptible to these complications and complicate their management significantly. Approximately 6% of trauma patients experience HAIs, with ventilator-associated pneumonia (VAP) being the most common kind [17]. Undiagnosed or mismanaged chronic comorbidities may exacerbate both short-term and long-term effects. These medical disorders, intensified or instigated by traumatic injuries and detrimental social circumstances, may mutually reinforce each other. Thus, an unacknowledged chronic fatal trifecta of trauma is created [18]. The possibility of discrimination against pre-existing infectious pathogens, which are not part of the body's normal flora and have been acquired during hospitalization, is particularly significant. Activating the pathogenicity of bacteria or fungi in the human microbiota under conditions of diminished immunity due to pre-existing pathology cannot be equated with the occurrence of an iatrogenic infection resulting from invasive procedures and/or breaches in the protocols for surveillance, prevention, and control of nosocomial infections. A problem resulting from an infection contracted during hospitalization or while receiving medical care may establish grounds for liability against the healthcare institution, potentially entitling the injured patient to compensation.

The autopsy findings may diverge from the clinically acknowledged causes of death in instances of fatal trauma. Various factors, such as pre-existing conditions, age, trauma mechanism, effectiveness of cardiac resuscitation, survival duration, and access to multi-slice spiral computed tomography (MSCT) or emergency diagnostics, all contribute to this disparity. However, the autopsy is the most reliable method for determining the cause of death and ensuring quality control in trauma-related fatalities, serving as the definitive medical procedure to evaluate treatment effectiveness and providing essential insights [19].

## Conclusions

In cases of intricate, multivariate pathology, forensic pathologists may face challenges in ascertaining the cause of death and the influence of the patient's other conditions on the determination of death. In such instances, a customized approach and a thorough assessment of the severity of traumatic injuries, of the patient's medical history, and of their clinical course while hospitalized are crucial. The aforementioned scenarios illustrate particular challenges, and we have aimed to outline our strategy for addressing them, allowing forensic doctors to objectively determine and express the cause of death, the manner of death, the causal relationship between traumatic injuries and death, and the influence of pre-existing conditions or HAIs on the fatal outcome.
